# Sodium *p*-Aminosalicylic Acid Reverses Sub-Chronic Manganese-Induced Impairments of Spatial Learning and Memory Abilities in Rats, but Fails to Restore γ-Aminobutyric Acid Levels

**DOI:** 10.3390/ijerph14040400

**Published:** 2017-04-10

**Authors:** Shao-Jun Li, Chao-Yan Ou, Sheng-Nan He, Xiao-Wei Huang, Hai-Lan Luo, Hao-Yang Meng, Guo-Dong Lu, Yue-Ming Jiang, Tanara Vieira Peres, Yi-Ni Luo, Xiang-Fa Deng

**Affiliations:** 1 Department of Toxicology, School of Public Health, Guangxi Medical University, Nanning 530021, China; lishaojun0613@163.com (S.-J.L.); oak009@163.com (C.-Y.O.); qiqishengnh@sina.com (S.-N.H.); xwhuang82@163.com (X.-W.H.); fanxingddlx@163.com (H.-L.L.); moso_17@163.com (H.-Y.M.); golden_lu@hotmail.com (G.-D.L.); ninimimimomo@126.com (Y.-N.L.); 2 Department of Toxicology, School of Public Health, Guilin Medical University, Guilin 541004, China; 3 Guangxi Colleges and Universities Key Laboratory of Prevention and Control of Highly Prevalent Diseases, Guangxi Medical University, Nanning 530021, China; 4 Key Laboratory of Early Prevention and Treatment for Regional High Frequency Tumor, Ministry of Education, Nanning 530021, China; 5 Department of Molecular Pharmacology, Albert Einstein College of Medicine, Forchheimer, 209, 1300 Morris Park Ave, Bronx, NY 10461, USA; Tanara.Peres-Vieira@einstein.yu.edu; 6 Department of Anatomy, School of Pre-Clinical Medicine, Guangxi Medical University, Nanning 530021, China; dengxfa@163.com

**Keywords:** sodium para-aminosalicylate, sub-chronic manganese exposure, spatial learning and memory ability, γ-aminobutyric acid, basal ganglia

## Abstract

Excessive manganese (Mn) exposure is not only a health risk for occupational workers, but also for the general population. Sodium para-aminosalicylic acid (PAS-Na) has been successfully used in the treatment of manganism, but the involved molecular mechanisms have yet to be determined. The present study aimed to investigate the effects of PAS-Na on sub-chronic Mn exposure-induced impairments of spatial learning and memory, and determine the possible involvements of γ-aminobutyric acid (GABA) metabolism in vivo. Sprague-Dawley male rats received daily intraperitoneal injections MnCl_2_ (as 6.55 mg/kg Mn body weight, five days per week for 12 weeks), followed by daily subcutaneous injections of 100, 200, or 300 mg/kg PAS-Na for an additional six weeks. Mn exposure significantly impaired spatial learning and memory ability, as noted in the Morris water maze test, and the following PAS-Na treatment successfully restored these adverse effects to levels indistinguishable from controls. Unexpectedly, PAS-Na failed to recover the Mn-induced decrease in the overall GABA levels, although PAS-Na treatment reversed Mn-induced alterations in the enzyme activities directly responsible for the synthesis and degradation of GABA (glutamate decarboxylase and GABA-transaminase, respectively). Moreover, Mn exposure caused an increase of GABA transporter 1 (GAT-1) and decrease of GABA A receptor (GABA_A_) in transcriptional levels, which could be reverted by the highest dose of 300 mg/kg PAS-Na treatment. In conclusion, the GABA metabolism was interrupted by sub-chronic Mn exposure. However, the PAS-Na treatment mediated protection from sub-chronic Mn exposure-induced neurotoxicity, which may not be dependent on the GABA metabolism.

## 1. Introduction

Manganese (Mn) is universally present in the body and it plays a vital role in several brain functions [1]. The physiological Mn level in the brain is 1–2 µg/g in dry weight [2,3]. Excessive brain Mn accumulation may cause manganism, which is characterized by an extra pyramidal motor disorder analogous to Parkinson’s disease (PD) [4,5], such as cognitive deficits and psychiatric disturbances [6]. Neuroimaging studies have demonstrated that the Mn-induced extra pyramidal motor disorders were associated with the disruption of basal ganglia circuitry, especially *globus pallidus*, *substantianigra pars reticulate*, and *striatum* [4,5].

Although the molecular mechanisms of Mn-induced neurotoxicity have yet to be delineated, initial studies have implicated that Mn impaired dopamine (DA) homeostasis through the deregulation of transcription and protein levels of DA receptors and transporters [7,8]. Recently, Mn exposure was further associated with changes in other neurotransmitters, such as γ-aminobutyric acid (GABA) levels in striatum [4,9,10]. GABA, as the most widespread inhibitory neurotransmitter in the brain, plays a key role in regulating the excitatory signals of the motor function in the basal ganglia [11,12,13]. It is synthesized from glutamate (Glu) via decarboxylation by Glu decarboxylase (GAD); but degraded by GABA-transaminase (GABA-T). Mn preferentially accumulates in the basal ganglia, where GABA projections are enriched [4]. Studies in non-human primates have found that the Mn-induced cognitive deficits were related to an alteration of the GABA level and disruptions in the glutamine(Gln)/Glu-GABA cycle (GGC) between astrocytes and neurons in the prefrontal cortex [14,15]. Lai and other investigators reported that short-term Mn exposure (6–20 mg/kg MnCl_2_ i.p. for a month) decreased GAD and/or GABA-T levels in the striatum [16], overall brains [17], and globus pallidus, but not the striatum, of aged rats [18,19]. Taken together, these studies proposed an important role of GABA metabolism in the Mn-induced behavioral deficit.

In addition to initial therapeutic attempts using levodopa [20] and EDTA [21], clinical interventions using sodium *p*-aminosalicylic acid (PAS-Na) were shown to be therapeutically efficacious in manganism patients [22,23]. PAS-Na could promote Mn excretion in experimental animal models [24,25]. Apart from its chelating effects, whether PAS-Na can restore the Mn-induced neurotoxicity through GABA metabolism in vivo has yet to be determined. Thus, the present study aimed to determine whether PAS-Na has protective effects in the sub-chronic Mn exposure-induced spatial learning and memory impairments, and investigated the possible involvements of the GABA metabolism.

## 2. Materials and Methods

### 2.1. Ethics Statement

All of the experimental procedures were performed in strict accordance with the principles and guidelines of the National Institutes of Health Guide for the Care and Use of Laboratory Animals. The experimental protocol were evaluated and approved by the Animal Use and Care Committee of Guangxi Medical University (ethical approval No. 201412001).

### 2.2. Experimental Animals

Sprague-Dawley (SD) male rats, of a specific pathogen-free grade (SPF), were purchased from the Experimental Animal Center of Guangxi Medical University (SCXKG 2009-0003, Nanning, China). The animals were housed at 24 ± 1°C, 55% ± 10% humidity, with food and water *ad libitum*.

### 2.3. Experimental Design

**Figure 1:**
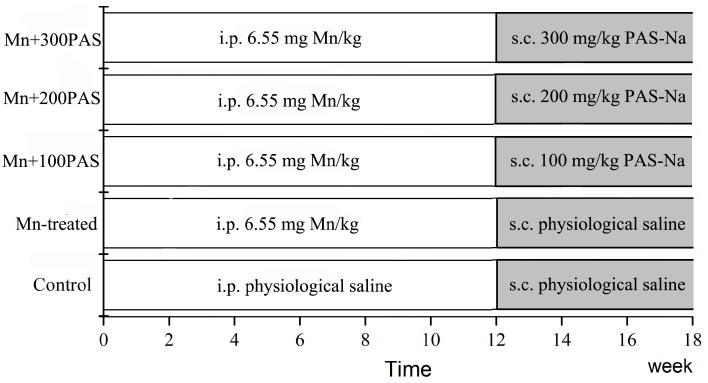
Experimental design.

After the animals had acclimated for one week, sub-chronic exposure was performed. Fifty male SD rats (80 ± 20 g, 4-weeks-old) were divided randomly into five groups (with 10 rats in each groups), including control, Mn-treated, and three doses of PAS-Na (100, 200, and 300 mg/kg) treatment (Mn + 100, Mn + 200 and Mn + 300 PAS) groups. As in Figure 1, the Mn-treated, Mn + 100, Mn + 200, and Mn + 300 PAS groups received intraperitoneal (i.p.) injections of MnCl_2_ (as 6.55 mg/kg Mn) once a day, five days per week, for 12 weeks, before subsequent treatment with physiological saline (for the Mn-treated group) and different doses of PAS-Na (100, 200, and 300 mg/kg, subcutaneous (s.c.) injections) in the back area of rats once a day, five days per week, for an additional six weeks. The control group received sterile physiological saline in the meantime. All of the i.p. and s.c. injections were given at volumes of 0.2 mL per 100 g rat weight. MnCl_2_ and PAS-Na were dissolved in sterile physiological saline each day before the injections.

The dosing regimen i.p. injection of MnCl_2_ (as 6.55 mg/kg Mn) was chosen based on previous experiments [26,27]. The dosing regimen of the PAS-Na treatment chosen in the present study was based on the dosage conversion, according to FDA protocol (Guidance for Industry and Reviewers Estimating the Safe Starting Dose Clinical Trials for Therapeutics in Adult Healthy Volunteers, 2011). Clinically, PAS-Na is administrated at 2–6 g per day through intravenous (i.v.) infusion to patients with manganism [23,28]. The conversion formula was established accordingly: Dose used in rats = Dose used in human/normal human body weight × 0.018 (the dose modifying factor)/0.2 kg (rats’ body weight). Therefore, the calculated dosage regime for mice was 180–540 mg/kg. Moreover, it has been reported that the LD_50_ of PAS in mice is 6811 mg/kg via i.p. injections [29,30]. Based on the calculation, we thus chose 100, 200, or 300 mg/kg PAS-Na to prevent Mn-induced neurotoxicity.

The PH value of the MnCl_2_ solution was between 5.5 and 5.9, and was thus adjusted to 6.3 by using 1 mol/L NaHCO_3_ solution before administration. The PH value of the PAS-Na solution was between 6.7 and 7.0. It is worth noting that the salt form of PAS-Na is not as acidic as PAS. We did not found any observable signs of irritation in the sites of injections throughout drug administration.

### 2.4. Morris Water Maze (MWM) Test

The MWM test was performed within 24 h after the final administration, in order to assess the spatial learning and memory ability of the experimental animals according to previous studies [31]. The apparatus consisted of a water pool (210 cm diameter × 60 cm height, and colored with a black non-toxic dye). The pool was separated into four quadrants, with four equally spaced points around the edge designated as north (N), east (E), south (S), and west (W) with different shapes of white board. A black-colored round platform (2 cm below the water surface) was placed in the center of the S quadrant of the pool. The MWM test was performed in two phases: training trials and spatial probe trials. Before the training, rats were placed on the platform for 15 s, in order that they could familiarize themselves with the environment. Then, the rats were trained to navigate the submerged platform for five consecutive days, from different locations (N, E, S, and W). Rats were allotted 90 s to reach the platform. The trials were terminated once the rats reached the platform. Those that failed to find the platform within 90 s were directed to the platform and placed on the platform for 15 s. The escape latency and swimming distance were recorded. On the sixth day, the platform was removed for the spatial probe trial. The number of platform crosses and the swimming speed were recorded during a period of 120 s.

### 2.5. Tissue Extraction and Preparation

At the end of the experiment, the rats were euthanized by cervical dislocation and the basal ganglia were extracted and immediately frozen in liquid nitrogen, before being stored at −80 °C until analysis.

### 2.6. Determination of GABA Levels

GABA levels in the basal ganglia were measured by using high performance liquid chromatography (HPLC) analysis, as previously described [31,32]. The total protein concentrations in the lysates were determined with a BCA kit (Beyotime Laboratories Inc., Shanghai, China). The results were expressed as the content of GABA μmol/g protein.

### 2.7. Determination of Glu Decarboxylase (GAD) Activity

The enzymatic activity of GAD (as the main enzyme for GABA synthesis) was measured as previously described [9], with minor modifications. The basal ganglia (30–50 mg) were weighed and homogenized in 100 μL cold homogenization buffer (containing 30 mM HEPES, 0.1% Triton X-100, and 5% glycerol). After centrifugation at 10,000× *g* for 5 min at 4 °C, the supernatants were divided into two tubes. Pyridoxal-5-phosphates, 5 mM Glu, and 100 μM gabaculine were added to both tubes. One tube was reacted for 30 min at 37 °C with constant shaking; the other tube was stopped immediately upon the addition of perchlorate buffer (contains 0.5 mol/L acetic acid, 0.5 mol/L sodium acetate, and 0.4 mol/L sodium perchlorate) to the supernatant. After centrifugation, the supernatant was analyzed by HPLC [31,32]. The GAD activity was calculated as the reaction rate of the newly synthesized GABA (total GABA level in the reaction tube minus the original GABA level), and was then normalized to the total protein concentrations in the lysates. The results were expressed as μmol/h.g brain protein.

### 2.8. Determination of GABA-Transaminase (GABA-T) Activity

The enzymatic activity of GABA-T (as the main enzyme to metabolize GABA) in the basal ganglia was determined with a coupled enzyme assay, as previously reported [9]. Briefly, the basal ganglia were weighed and homogenized with 100 μL cold homogenization buffer (contains 3 mM HEPES, 20 mM KCl, 2.5 mM sucrose, 500 μM EDTA and 5 mM MgCl_2_, pH 7.5), and were then centrifuged at 800× *g* for 10 min and 30,000× *g* for 20 min. The resultant precipitate was re-suspended with buffer (0.1% Triton X-100, 30 mM HEPES (pH 7.5), 5% (*v*/*v*) glycerol). Next, 15 μL suspensions were mixed with 285 μL buffer containing 100 mM pyrophosphate, 3.5 mM beta-mercaptoethanol, 1 mM NAD^+^, and 1 mM pyridoxal phosphate. The reaction was started by adding 10 μL of 5 mM α-ketoglutarate and 18 mM GABA for half an hour at 37 °C, and the fluorescence was measured with a fluorescence spectrophotometer (RF-5301 PC, Japan, λex = 255 nm, λem = 459 nm). The blank consisted of 25 μL deionized water, plus 285 μL 100 mM pyrophosphate buffer. The results were expressed as IU/g brain protein.

### 2.9. Real-Time Polymerase Chain Reaction (RT-PCR) and Western Blot Analysis

The mRNA and protein expression of the GABA A receptor (GABA_A_) and GABA transporter 1 (GAT-1) in the basal ganglia were analyzed by RT-PCR and western blotting, respectively, as previously described [31]. The tissues were homogenized on ice with the RIPA buffer (150 mM NaCl, 50 mM TrisHCl pH 7.4, 1 mM EDTA, 1% Triton X-100, 0.5% DOC, and 0.1% SDS), with the addition of protease inhibitors (1 mM phenylmethylsulfonyl fluoride, 10 μg/mL pepstatin A, 10 μg/mL leupeptin, 10 μg/mL aprotinin). After 30 min in an ice bath, the tissue homogenate was centrifuged at 10,000× *g* for 15 min at 4 °C. The expressions of GAPDH were used to normalize the expression of the target genes and proteins. The total protein concentrations in the lysates were determined with the BCA kit. The results of RT-PCR were expressed as the % of control, while the results of Western Blot were expressed as the relative density of GABA_A_ or GAT-1/GAPDH.

### 2.10. Statistical Analysis

Statistical analysis was performed with SPSS 16.0 for Windows (SPSS, Inc., Chicago, IL, USA). Data are expressed as mean ± SD. Since parametric statistical tests were used, Kolmogorov-Smirnov and Levene’s tests were used to evaluate the normality of distribution and homogeneity of variance of the data, respectively. Next, statistical differences in the escape latency and swimming distance were determined by repeated measure ANOVA, with Tukey’s *post-hoc* test for an analysis of differences between multiple sets of data. One-way ANOVA and Tukey’s *post-hoc* tests were used for analyzing other parameters (including probe trial test, GABA levels, GAD and GABA-T activity, mRNA and protein expression of GABA_A_ and GAT-1). If the *p-*value was <0.05, the result was considered statistically significant.

## 3. Results

### 3.1. PAS-Na Treatment Recovered Sub-Chronic Mn Exposure Induced Impairments of Spatial Learning and Memory Abilities

**Figure 2:**
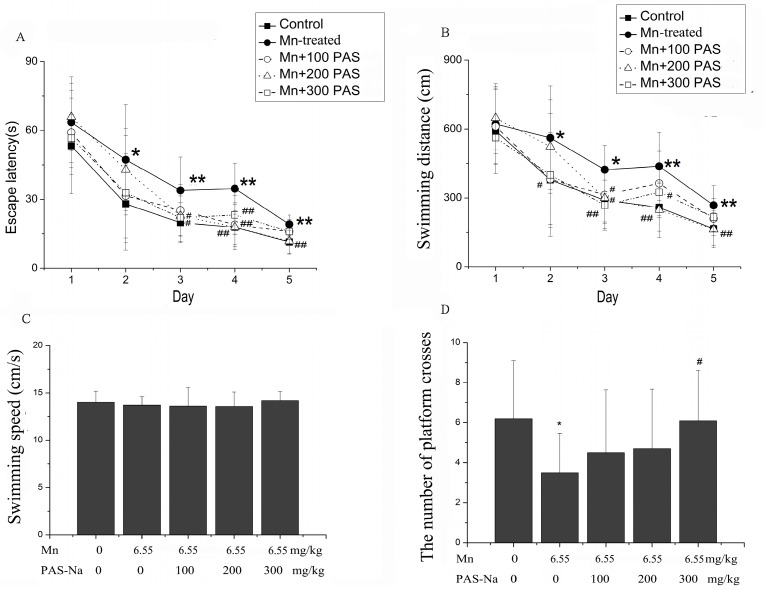
PAS-Na reverted the increase of escape latency and swimming distance induced by sub-chronical Mn exposure. (**A**) Escape latency; (**B**) Swimming distance; (**C**) Swimming speed; (**D**) The number of platform crosses. Data represent mean ± SD. N = 10 per group. * *p* < 0.05 or ** *p* < 0.01: significant as compared to control; # *p* < 0.05 or ## *p* < 0.01: significant as compared to the Mn-treated group.

As shown in the spatial navigation test for assessing learning abilities, Mn-treated rats showed learning impairments that were characterized by an increased escape latency and swimming distance, when compared with those of the control group (repeated measures ANOVA F(4, 45) = 3.382, *p* = 0.017 for escaping latency; and F(4, 45) = 3.505, *p* = 0.014 for swimming distance (Figure 2A,B). In contrast, PAS-Na treatments (100, 200, or 300 mg/kg) significantly improved the Mn-induced learning impairments, by decreasing the escape latency and the swimming distance (Tukey’s *post-hoc* test, *p <* 0.05 and *p <* 0.01 respectively, Figure 2A,B). On the other hand, in the spatial probe trial for assessing memory, the number of platform crosses of the Mn-treated group was less than those of the control group (3.50 ± 1.96 vs. 6.20 ± 2.28 s, one-way ANOVA, F = 3.278, *p* = 0.021, Figure 2C), suggesting memory deficits. As expected, PAS-Na treatment (300 mg/kg) also significantly attenuated the memory deficits induced by sub-chronic Mn exposure, restoring the number of platform crosses to control levels (multiple comparisons, *p* = 0.023, Figure 2D). Furthermore, there was no difference observed in the swimming speed between different groups (one-way ANOVA, F = 0.538, *p* = 0.677, Figure 2C), indicating that the aforementioned impairments by Mn did not result from possible muscular side effects of the Mn injections.

### 3.2. PAS-Na Treatment Failed to Restore Mn-Induced Decrease in GABA Levels in the Basal Ganglia

**Figure 3:**
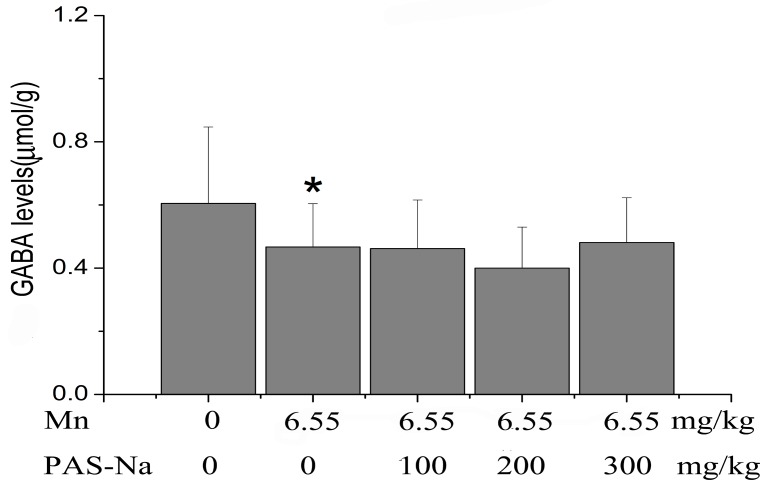
PAS-Na failed to reverse the Mn-induced decrease of GABA levels in the basal ganglia. Data represent mean ± SD. N = 5 per group. * *p <* 0.05: significant as compared to control.

Next, we measured the GABA level in the basal ganglia to determine the possible involvement of GABA metabolism. Sub-chronic Mn exposure for 12 weeks and a six-week exposure cessation led to decreased GABA levels compared with the control [F(4, 45) = 4.337, *p* = 0.003, multiple comparisons, *p* = 0.02, Figure 3]. In contrast to the protective effects of PAS-Na in Mn-induced neurotoxicity, all doses of PAS-Na treatment for six weeks failed to affect the Mn-induced changes in the GABA levels (multiple comparisons, *p* > 0.05).

### 3.3. PAS-Na Treatment Restored Mn-Induced Changes of GABA Enzymes

**Figure 4:**
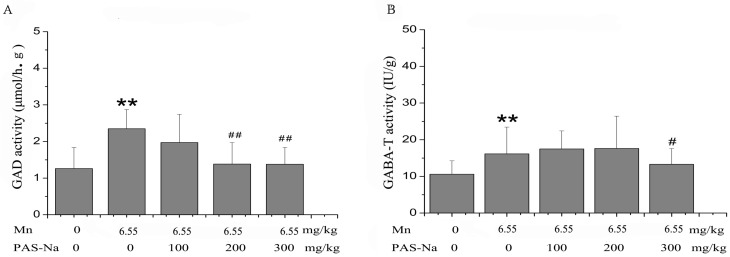
PAS-Na attenuated the increase of GAD and GABA-T activity in the basal ganglia of Mn-exposed rats. (**A**) GAD activity; (**B**) GABA-T activity. Data represent mean ± SD. N = 5 per group. ** *p <* 0.01: significant as compared to control; *# p <* 0.05 or *## p* < 0.01: significant as compared to Mn-treated group.

To further investigate the effects of Mn on GABA metabolism, the enzymatic activities of GAD (a GABA-synthesizing enzyme) and GABA-T (a GABA-degrading enzyme) in the basal ganglia were analyzed. After Mn exposure for 12 weeks and a six-week exposure cessation, the GAD activity in the Mn-treated group was significantly increased compared with the control [F(4, 45) = 5.061, *p* = 0.003, and multiple comparisons, *p* = 0.0005, Figure 3]. In a similar pattern, the GABA-T activity of the Mn-treated group was significantly increased compared with the control [153% of control, F(4, 45) = 4.261, *p* = 0.004, and multiple comparisons, *p* = 0.0002, Figure 4B]. In contrast, treatment with either 200 or 300 mg/kg PAS-Na for six weeks caused significant reductions in GAD activity, to 41.06% and 41.31% of the Mn-treated group, respectively(multiple comparisons, *p* = 0.005 or 0.009 respectively, Figure 4A). PAS-Na treatment at the highest dose (300 mg/kg, but not 100 or 200 mg/kg) for six weeks also significantly reduced GABA-T activity by 24.0%, compared with the Mn-treated group (multiple comparisons, *p* = 0.005, Figure 4B).

### 3.4. PAS-Na Prevented Mn-Induced Transcriptional Changes in GABA_A_ and GAT-1 Expression

**Figure 5:**
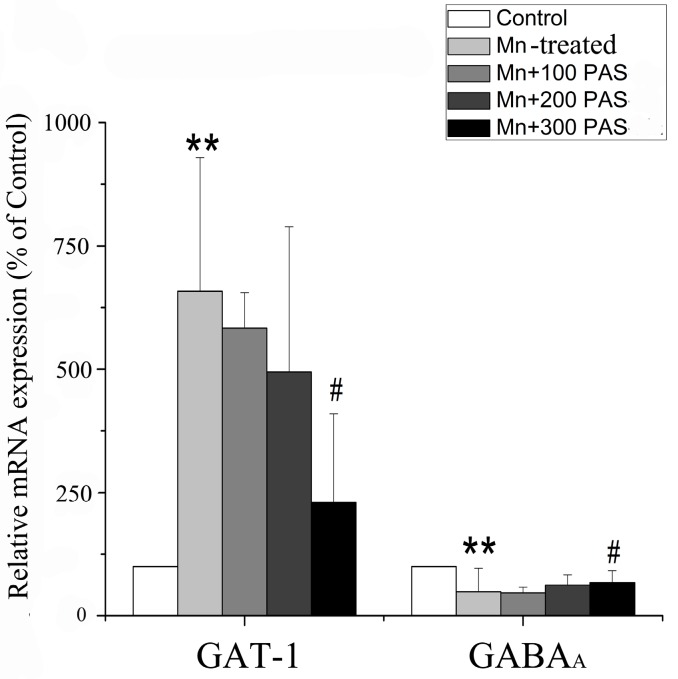
PAS-Na attenuated the changes of GABA_A_ and GAT-1 in the transcription level in the basal ganglia of Mn-exposed rats. Data represent mean ± SD. N = 5 per group. ** *p <* 0.01: significant as compared to Control; # *p <* 0.05: significant as compared to Mn-treated group.

**Figure 6:**
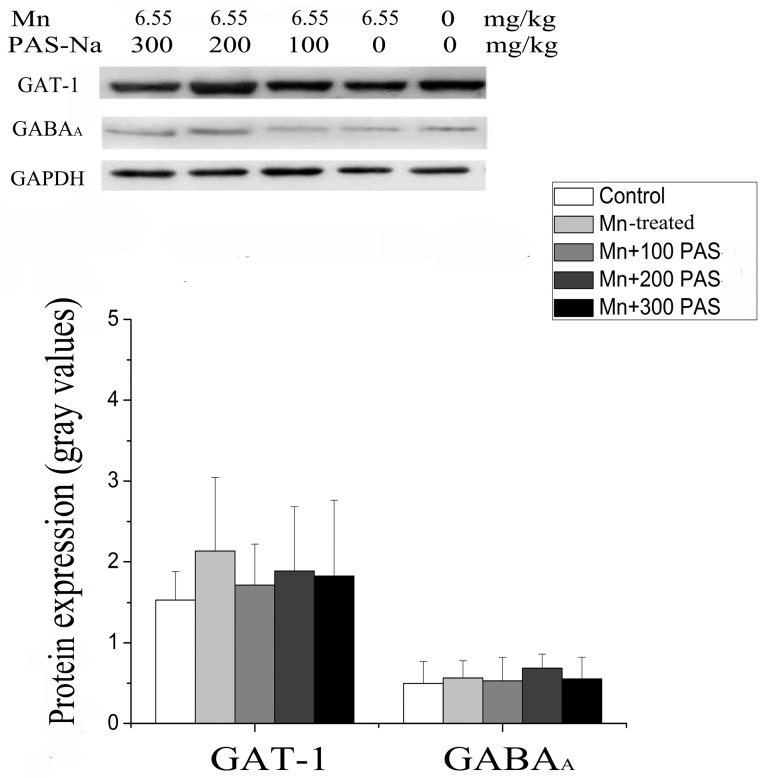
Mn exposure did not alter the GABA_A_ and GAT-1 protein expression in the basal ganglia. Data represent mean ± SD. N = 5 per group.

To investigate whether Mn affected GABA metabolism through the deregulation of its receptor or transporter, the mRNA and protein expressions of GABA_A_ and GAT-1 in the basal ganglia were measured. Sub-chronic Mn exposure decreased GABA_A_ mRNA expression in the basal ganglia [F(4, 20) = 12.182, *p* = 0.0008, multiple comparisons, *p* = 0.007, left panel of Figure 5], but significantly increased GAT-1 mRNA levels [F(4, 20) = 12.182, *p* = 0.003, multiple comparisons, *p* = 0.0002, right panel of Figure 5], when compared with the control. Treatment with 300 mg/kg PAS-Na (but not 100 and 200 mg/kg) for six weeks restored the above-mentioned changes by increasing GABA_A_ mRNA expression (multiple comparisons, *p* = 0.033) and decreasing GAT-1 mRNA expression (multiple comparisons, *p* = 0.006). Although it seemed that sub-chronic Mn exposure increased GAT-1 protein expression and PAS-Na treatment could recover this, all of the changes failed to reach statistical significance (*p* > 0.05, Figure 6).

## 4. Discussion

Mn, as an essential metal element, is normally present as inorganic soluble compounds in drinking water, diet, and airborne particles, such as gasoline adjuvant methylcyclopentadienyl Mn tricarbonyl and the fungicide maneb Mn ethylene-1,2-bisdithiocarbamate etc. [33,34]. Occupational Mn exposures are commonly achieved through the respiratory tract, digestive tract, and skin contacts, while environmental exposure is due to ingestion and inhalation [3]. The present study aimed to investigate the molecular mechanisms of Mn-induced neurotoxicity and the protective effects of PAS-Na. Thus, we chose i.p. injections, a non-physiological route of administration, to establish the Mn-induced neurotoxicity, because of the following reasons. Firstly, the dose of Mn could be accurately administrated through i.p. injection. In contrast, if administrated through ingestion and inhalation, individual rats may have received a variable total dosage, which may lead to higher levels of standard deviations in the final results [35]. Secondly, several publications conducted in our laboratory and others confirmed that i.p. injections of Mn could successfully establish the animal model of Mn-induced neurotoxicity [22,25,36]. These results suggest that inorganic Mn could pass through the blood-brain-barrier and cause neurotoxicity, despite the different administration routes.

At the levels of Mn exposure used in the present study, impairments of learning and memory abilities were observed, along with a disrupted GABA metabolism. The blood Mn level achieved after sub-chronic Mn exposure was 53.06 ± 19.54 μg/L, which was much higher than those of the control (7.2 ± 3.49 μg/L, data not shown). The dosage of Mn was not exceedingly high, if compared with the results from previous publications. Firstly, environmental or occupational Mn exposure can cause an accumulation as high as 88.0 μg/L in human blood. In studies conducted in central Mexico, the blood Mn levels in the general population ranged from 7.5 to 88.0 μg/L [37,38,39]. Another French study also reported that half of the cord blood samples had Mn concentrations greater than 40 μg/L, ranging from 14.9–92.9 μg/L (*n* = 112) [40]. Secondly, animal studies confirmed that sub-chronic Mn exposure could increase Mn concentrations to as high as those in humans. Two non-human primate studies using cynomolgus macaque monkeys reported that 14-weeks administration of Mn caused it to accumulate in the blood, reaching a level of 89.9 ± 10.5 μg/L (ranging from 63.5 to 134.1 μg/L, vs. 10.8 ± 5.0 μg/L in the control group), and 28-weeks administration of Mn led to a value of 70.8 ± 15.6 μg/L (ranging from 33.3 to 118.8 μg/L) [26,27]. Moreover, it has been reported that the LD_50_ of Mn in mice is 89 mg/kg (as 320 mg MnCl_2_/kg) via i.p. injections [30]. Lastly, the dosing regimen chosen in the present study and the consequent neurotoxicity effects were in line with previous publications conducted in the laboratory [31,41]. Therefore, the dosage of Mn exposure in our rat model was within the reasonable range according to previous human and animal findings.

Clinically, PAS-Na was administrated through intravenous (i.v.) infusion in patients of manganism and achieved good prognoses [23,28]. While PAS is slowly and relatively completely absorbed [42], the short blood half-life (t1/2: 2–3 h) indicates that PAS is quickly removed from the systemic circulation, leaving a relatively low blood level [43]. To achieve the therapeutic effect in manganism, the drug is usually given by intravenous infusion at 2–6 g per day [23,28]. A long time and highly sustainable blood level following a high dose of PAS may allow sufficient PAS molecules to pass across the brain barriers, thus being able to mobilize and remove Mn from its intracellular depots. However, an intravenous injection may not be suitable for the rat model because rat models are unsuitable for long periods of intravenous infusion. Thus, subcutaneous injections of PAS-Na were chosen in the present study, because of the following reasons. Firstly, PAS-Na was given subcutaneously at the back of rats, different from the i.p. sites of Mn, to avoid possible in situ chemical interactions. Secondly, it is unfeasible to undertake i.v. infusion in the irritable rats. In a toxicological experiment, a subcutaneous injection of PAS-Na was widely used for its practical simplicity. Lastly, we found that, under these routes, there was a significant decrease in the Mn levels of sub-chronic Mn exposure after 160 mg/kg PAS-Na treatment (decreased to 29.0 ± 17.4 μg/L from 53.06 ± 19.54 μg/L, data was not shown here), which confirmed the previous animal studies conducted in our laboratory and others [22,25,36]. For the sub-chronic treatment of PAS-Na for six weeks, the slow absorption rates of PAS-Na through subcutaneous injections could be neglected. Additionally, the present study showed that PAS-Na treatment effectively restored Mn-induced impairments of spatial learning and memory abilities, suggesting that the rat model was successful.

GABA, as a major inhibitory neurotransmitter in the mammalian CNS, plays an important role in mediating psychological health and cognitive function. Tadashi and Tomoko [44] found that GABA levels were decreased in the spinal cord of PD and epilepsy patients. A ^1^H-magnetic resonance spectroscopy study showed that decreased GABA levels in the anterior cingulated cortex/medial prefrontal cortex were related with psychiatric disorders (Panic disorder) [45]. More importantly, the changes in the GABA levels in the basal ganglia were associated with alterations of the cognitive and memory abilities in PD patients [46,47]. The involvement of GABA in Mn-induced toxic effects has been previously proposed [48]. However, the effects of excessive Mn exposure on GABA metabolism in the literature are conflicted. For example, Bonilla and colleagues reported that chronic Mn exposure increased GABA levels in rodent striatum [49], which was corroborated by others [4,10,32]. However, a myriad of studies has shown contradictory results [50,51]. The present study indicated that sub-chronic Mn exposure impaired spatial learning and memory abilities (Figure 2) and disrupted GABA metabolism (Figure 3) in rats. The controversial effects of Mn on GABA homeostasis may be due to the diverse species used, experimental methods of GABA determination, routes of Mn exposure, and the Mn exposure dose and periods. Unexpectedly, although PAS-Na treatment restored the learning and memory deficits induced by sub-chronic Mn exposure, it failed to restore the decrease of GABA levels in the basal ganglia.

Because exogenous GABA cannot cross the blood-brain barrier, the brain GABA is synthesized from Glu by GAD and metabolized by GABA-T [19]. Although a few in vivo studies indicated that GAD activity failed to directly contribute to the changes of GABA levels in the same brain regions affected by Mn [9,52], several other studies found that Mn exposure directly affected these two enzymes. For example, the decreases of GAD and GABA-T levels in the brain were found subsequent to i.p. injections of MnCl_2_ [17]. Tomas-Camardiel et al. also found that Mn exposure significantly decreased GAD mRNA levels in the *globus pallidus*, but not in the striatum of aged rats [18]. However, the present study showed a conflicting effect that sub-chronic Mn exposure increased both GAD and GABA-T activity in the basal ganglia (Figure 4). Considering the overall decrease of GABA levels after Mn exposure (Figure 3), the increased activity of GABA-T (a GABA-degrading enzyme) may overwhelm that of GAD (a GABA-synthesizing enzyme). These results may be inconsistent with previous publications, which might arise from the different brain regions analyzed, the different ages of the animals used, and different Mn exposure doses and periods. This illustrates how Mn intoxication can cause specific effects that vary according to the model of intoxication being used.

The mechanisms of Mn-induced disruption in the GGC between astrocytes and neurons have been well reviewed, e.g., protein kinase C (PKC) that can down-regulate Glu transporters [53]. It has been reported that Mn exposure increased PKC activity, PKC-*δ* phosphorylation, and its interaction with Glu transporter. Moreover, both GABA_A_ and GABA_B_ receptor proteins or mRNA expression were found to be decreased in *Globus pallidus*, but increased in *Substantia nigra*, of patients with neurodegenerative conditions, including Alzheimers’s disease, PD, and multiple sclerosis [54]. Animal models lacking GABA_B_ (which causes anxiety and nervousness) and GAT-1 (which causes tremor and anxiety) confirmed their involvement in neurodegenerative diseases [55,56]. Andersonand his colleagues found that Mn exposure altered both GABA transporter (GAT-1) and receptor (GABA_A_ and GABA_B_) protein expressions [57,58]. The current study demonstrated that sub-chronic Mn exposure increased GAT-1 mRNA, but decreased the GABA_A_ mRNA in the basal ganglia. However, their protein expressions were not consistent with the changes of mRNA. There are three possible reasons for the inconsistencies between mRNA expression and protein expression. Firstly, the turnover expression level of protein is influenced by both protein syntheses from mRNA and protein degradation through proteasome. Although we showed the alteration of mRNA, we cannot rule out that the protein degradation of these two proteins were affected in the same way. In a similar pattern, the GABA level in Figure 3 was unchanged, because of the consistent increase in the activities of both the GABA-synthesizing enzyme GAD and GABA-depredating enzyme GABA-T (Figure 4). Secondly, the semi-quantification method of Western-blotting by using gray values may underestimate the protein changes. In contrast, qRT-PCR using fluorescent dyes can differentiate the small changes of mRNA expression. But again, we cannot rule out technical error like over-exposing GAT-1 signals. Thirdly, we pooled the samples to do subsequent experiments because of the small size of the basal ganglia. The reasons why the results of mRNA and protein expression were not consistent may be due to the fact that we used different samples to determine the mRNA and protein expression, which was similar to the results of the previous studies [58].

Mn-induced PD patients appeared irresponsive to L-dopa treatment [59,60], and thus L-dopa is no longer prescribed in clinical treatments for Mn intoxication. Tandon et al. were the first to demonstrate that PAS efficiently promoted Mn excretion, which was confirmed by subsequent studies in other labs [24,25,36]. PAS-Na is a low toxic antidote. Our previous in vitro study showed that PAS-Na alone did not cause cell injuries and any changes in the amino acid neurotransmitter in the primary-cultured basal ganglia neurons and astrocytes [32,61]. Additionally, Zheng et al. (2009) also showed that PAS alone has no effects on trace element levels [25] and our later in vivo study also did not find PAS-Na alone to have effects on the spatial learning and memory ability. However, the mechanisms associated with PAS-Na’s efficacy have yet to be determined. Its protective effects have been ascribed to its ability to chelate Mn and promote its excretion [25,62]; restore the alteration of iron levels induced by Mn [25]; inhibit inflammatory reactions and oxidative stress [63]; and restore Mn-impaired choline acetyltransferase activity in neurons [22]. Recently, our laboratory found that PAS-Na might play an antagonistic role in the imbalance of Mn-impaired neurotransmitters [32]. An in vivo study demonstrated that PAS-Na treatment restored the sub-acute Mn exposure-induced increase of the Glu/GABA ratio [31]. The results of this study indicated that although PAS-Na treatment for six weeks restored the alterations of GAD, GABA-T activity induced by Mn, PAS-Na failed to recover the decreased GABA levels in the basal ganglia of Mn-exposed rats. The similar patterns of decrease in both GAD and GABA-T activities by PAS-Na treatment may explain why PAS-Na failed to affect the overall GABA level. These results may also suggest that PAS-Na could restore the Mn-induced impairments of spatial learning and memory ability, independent of GABA.

## 5. Conclusions

In summary, our results demonstrated that sub-chronic excessive Mn exposure impaired spatial learning and memory abilities, which could be effectively restored by PAS-Na treatment. The GABA metabolism is involved in Mn-induced neurotoxicity. However, PAS-Na failed to recover the Mn-induced alteration of GABA levels in the basal ganglia. We cannot rule out the possible indirect effects of PAS-Na on Mn-induced interruptions of GABA metabolism, since PAS-Na treatment reversed Mn-induced alterations in the enzymes directly responsible for the synthesis and degradation of GABA, and the GABA receptor and transporter mRNA expression. Although we fail to find the involvement of GABA metabolism in the efficacy of PAS-Na on Mn-induced neurotoxicity, these results provide novel evidence in support of the efficacy of PAS-Na treatment on Mn-induced spatial learning and memory impairments. The possible effects of PAS-Na on Mn-induced GABA metabolism unbalance need to be further studied.
